# A new 3D-method to assess the inter implant dimensions in patients – A pilot study 

**DOI:** 10.4317/jced.56557

**Published:** 2020-02-01

**Authors:** Alexander Schmidt, Jan-Wilhelm Billig, Maximiliane A. Schlenz, Bernd Wöstmann

**Affiliations:** 1 Dr Med Dent. Postdoctoral Researcher, Department of Prosthodontics, Dental Clinic, Justus-Liebig-University, Giessen, Germany; 2 Dr Med Dent. Assistant Researcher, Department of Prosthodontics, Dental Clinic, Justus-Liebig-University, Giessen, Germany; 3 Dr Med Dent. Professor and Head, Department of Prosthodontics, Dental Clinic, Justus-Liebig-University, Giessen, Germany

## Abstract

**Background:**

Complex implant treatments have steadily increased within implant prosthodontics. Based on the lower implant mobility, implant impressions need high accuracy in the model transfer to receive a high passive fit within the final prosthodontic restoration. To analyze the accurate 3-dimensional (3D) inter-implant-positions, a reference point is indispensable. However, there is no reference in the patients mouth, so the aim of the present study was to develop a new method based on a custom-made-measuring-aid (CMA) to assess the inter implant dimensions (InID) in patients.

**Material and Methods:**

Initially an implant master model (IMM/patient equivalent) was digitized by computed tomography. A CMA was fixed on the impression posts and the inter implant dimensions (InID) were recorded with a coordinate measurement machine (CMM). For comparison to conventional and digital impression techniques, 10 impressions per technique were taken. InIDs for the IMM, the CMA and the two impression techniques were compared. To give a proof of principle, the new 3D-method was applied to three patients as pilot cases. Results for trueness and precision were analyzed by pairwise comparisons (*p*< .05). All data were subjected to univariate ANOVA.

**Results:**

Mean deviation for InID ranged from 10.3±18μm(CMA) to 41.7±36μm(conventional). There were partially significant differences for InID between the CMA and the different impression techniques. There were no significant differences for InID within the CMA. The InID in the *in-vivo* evaluation ranged from 42.3μm to 376.7μm(digital) and from 58.3μm to 274.0μm(conventional). There were partially significant differences between the techniques.

**Conclusions:**

Within the limits of this study, with the developed method using a CMA it is possible to assess the true 3D-InID with a decisive higher accuracy than possible with a conventional or digital implant impression. Overall, the CMA in this study generated results that were deemed clinically useful for the investigated inter implant positions.

** Key words:**Dental Implants, Dimensional Measurement Accuracy, Dental Impression Technique, Intraoral Scanner.

## Introduction

In recent years, cases with complex implant treatments, such as “all-on-four”, are steadily increasing in implant prosthodontics (1). For their long-term success, a passive fit of the restoration is considered crucial (2,3). Thus, the accurate transfer of the implant position from the patient’s mouth to a plaster or virtual model is decisive. This need for precision is related to the finding that there is a ten-time lower implant mobility compared to the mobility of natural teeth (2). However, all impression methods available today, both conventional and digital, are inevitably prone to errors, which readily explains the high number of studies in this area (4-6). At closer inspection, nearly all of the investigations addressing this problem are limited to model-based in-vitro setups. Not a single method or clinical study could be identified that describes a possible way to assess the implant positions directly in the patients mouth and allows for a comparison to a conventional or digital model resulting from either a conventional impression or an intraoral scan. Therefore, the aim of this study was to develop a three dimensional method (3D-M) based on a custom made measuring aid (CMA) to precisely assess the three dimensional inter implant dimensions (InID) in patients. Therefore, the new method was first investigated in an *in-vitro* setup to avoid patient related influencing factors (e.g. saliva, movement). To give a proof of principle, the application of the presented method was applied to three patients as pilot cases.

The following hypothesis was tested: The 3D-M reproduces the three-dimensional inter implant dimensions (A: trueness; B: precision) decisively better than a conventional or digital impression and is therefore suiTable as a measuring technique for impression studies in patients.

## Material and Methods

The study was divided into an *in-vitro* test section, where the method was first examined in detail in a laboratory setup. This was followed by the application to three pilot cases. The investigation was conducted in full accordance with ethical principles, including the World Medical Association Declaration of Helsinki. The ethics committee of Justus Liebig University approved this study (re. no. 163/15).

**Figure 1:**
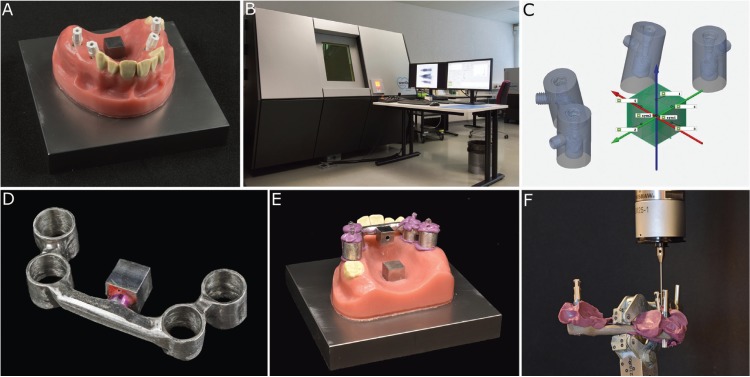
A: Implant master model (IMM) with tightened scanbodies on the premolar (15° inclination) and molar (0° inclination) implants and the reference cube in the middle of the palate. B: Multisensor coordinate measuring machine using X-ray tomography (TomoScope S). C: Reference file (patient equivalent) with the implant-abutment interface centers (IAICs) from the computed tomography (green points). D: Custom made measuring aid (CMA) with the inherent reference cube. E: CMA coded on the IMM. F: Measurement of the transferred CMA with tightened laboratory analogues using the coordinate measuring machine (CMM).

Based on a clinical case an upper jaw model made of a steel baseplate (100 × 100 mm) and a polymethylmethacrylate (PMMA) cover with four 3i Certain implants (Biomet 3i, Palm Beach Gardens, USA) was fabricated and served as an implant master model (IMM), representing the patient situation. For model stability, the implants were fixed in steel tubes placed on the right (upper right molar FDI 16; upper right premolar FDI 14) and left side (upper left molar FDI 26; upper left premolar FDI 24) in the premolar and molar region. AGC Cem served as the luting material (Wieland Dental, Pforzheim, Germany). The implants in the premolar area were inclined at 15° to the baseplate in the lateral direction. The implants in the molar region were positioned in parallel and at a 90° angle to the steel baseplate. A reference cube (10 × 10 × 20 mm) was positioned in the middle of the palate with its axis perpendicular to the baseplate (Fig. 1A).

To determine the position (center) of the four implant-abutment interface centers (IAICs) and the reference cube, the IMM was digitized with a multisensor coordinate measuring machine using X-ray tomography (TomoScope S running WinWerth, Werth Messtechnik, Giessen, Germany, linear accuracy <4 µm; Fig. 1B). The scan data were exported to a STL-file format and served as a reference file (patient equivalent; Fig. 1C).

For the production of the custom made measuring aid (CMA) four impression posts (IIIC41-molar region and IIIC42-premolar region, Biomet 3i, Palm Beach Gardens, USA) were tightened in the implants (10 Ncm) and undercuts were blocked out. An alginate impression was taken. Tubes were modeled around the impression posts with a circular distance of 1 mm around the implant impression posts, connected with bars (diameter 6 mm) and casted (cobalt-chromium-molybdenum). A further reference cube was attached to the CMA (Fig. 1D).

The CMA was coded with Impregum Penta (3MEspe, Seefeld, Germany) on the impression posts (Fig. 1E). After 20 minutes, the impression posts were unscrewed, and the CMA with the coded posts was removed from the model; then, four corresponding laboratory analogues were tightened on the impression posts (10 Ncm), and the CMA was mounted in a coordinate measurement machine (CMM, Thome Rapid, Messel, Germany, linear accuracy <3 µm).

**Figure 2:**
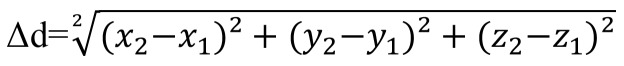
Calculation of the linear point to point deviations.

Thereafter, each implant position (i.e., laboratory analogue position) was assessed with the CMM (Fig. 1F). The entire procedure was repeated ten times. The data were exported into an IGES-format and imported into the GOM Inspect Software 2018 (GOM, Braunschweig, Germany). To calculate the linear point-to-point deviations in between the implants the coordinate system of the IMM and the CMA were superimposed and the deviation of the implant-abutment interface centers (inter implant dimension - InID) calculated via, (Fig. 2);

Δd= inter implant dimension (InID); x1,y1,z1= coordinates on the IMM; x2,y2,z2= coordinates on the CMA).

**Figure 3:**
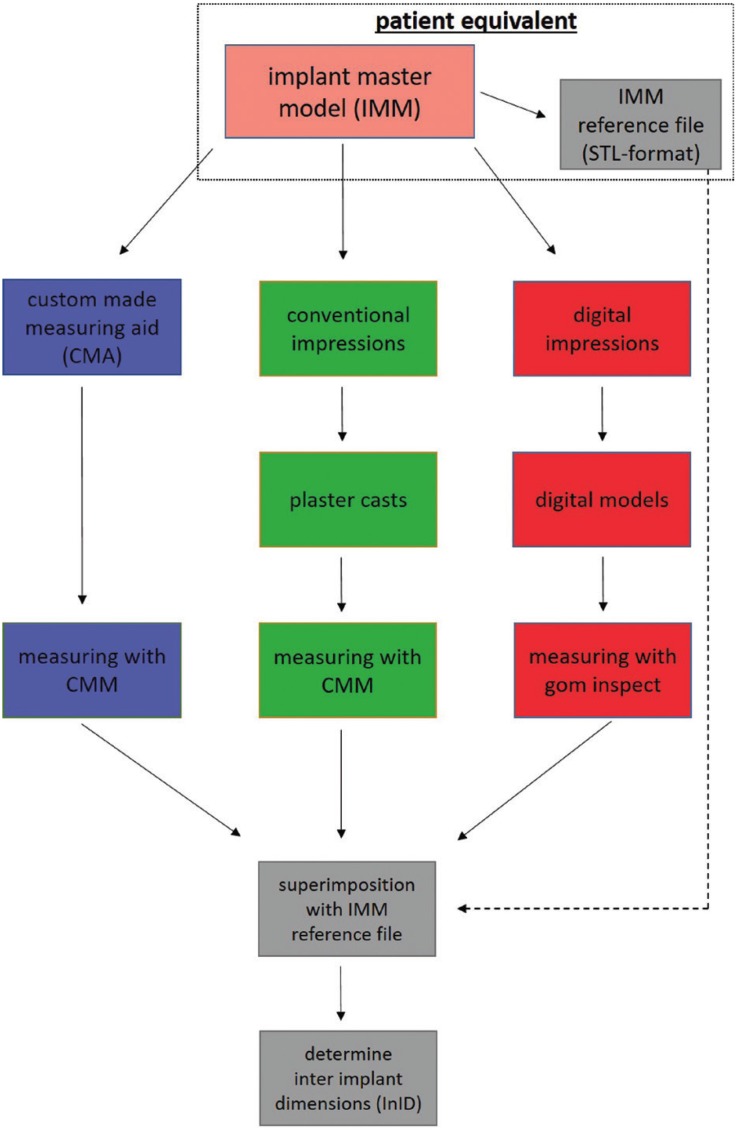
Overview of the entire workflow procedure.

After that, 10 conventional and 10 digital impressions were taken from the IMM. For a better overview, the entire workflow is depicted in Fig. 3. For the conventional impressions, four impression posts (IIIC41-molar region and IIIC42-premolar region, Biomet 3i, Palm Beach Gardens, USA) were tightened in the implants of the IMM (10 Ncm). Impressions were taken using a polyether material (Impregum Penta, 3M ESPE, Seefeld, Germany) with custom impression trays (a thickness of 3 mm) with a tubular design around the impression posts (open tray technique). After setting, the screws were subsequently untightened, and the impressions were removed from the IMM. Laboratory analogues (H-series, nt-trading, Karlsruhe, Germany) were attached to the impression posts and plaster casts were made with Fujirock EP (GC Corp., Tokyo, Japan). The casts were stored under laboratory conditions for 7 days. For the InID measurement of the plaster casts, four scanbodies (H-series, nt-trading, Karlsruhe, Germany) were tightened (10 Ncm) in the implant analogues and measured with a CMM (Thome Rapid, Messel, Germany). The data were exported in an IGES-format and imported to the GOM Inspect Software 2018 (GOM, Braunschweig, Germany).

For the digital impressions, the Trios 3 intraoral scanner (IOS; Trios3, software version 1.4.7.4, 3Shape, Copenhagen, Denmark) was used. Four scanbodies (H-series, nt-trading, Karlsruhe, Germany) were tightened in the IMM (10 Ncm), and the IOS was calibrated and scanned according to manufacturer’s specifications. The digital models were exported in a stl-format and imported to the GOM Inspect Software 2018 (GOM, Braunschweig, Germany).

The entire measurement procedure of the conventional and digital models was repeated ten times. The same examiner (J-W.B.) performed all experimental procedures.

For the *in-vivo* part, the methodology was investigated in a total of three patients as a pilot study. A new CMA was produced for each patient. The measurement was carried out in the same way as in the *in-vitro* part.

Verification of the normal distribution data was tested by Shapiro-Wilk and Levene’s tests. The results for the trueness and precision were analyzed by pairwise comparisons (*p*< .05). For the precision, a two-way factorial mixed ANOVA was used. For a better overview, the results of the *in-vitro* part were presented in boxplot format. The mean deviation describes the trueness, and the standard deviation depicts the precision. The results for the in-vivo part were presented in a bar graph. Statistical analysis was performed with SPSS 25 (IBM, Chicago, IL, USA).

## Results

**Figure 4:**
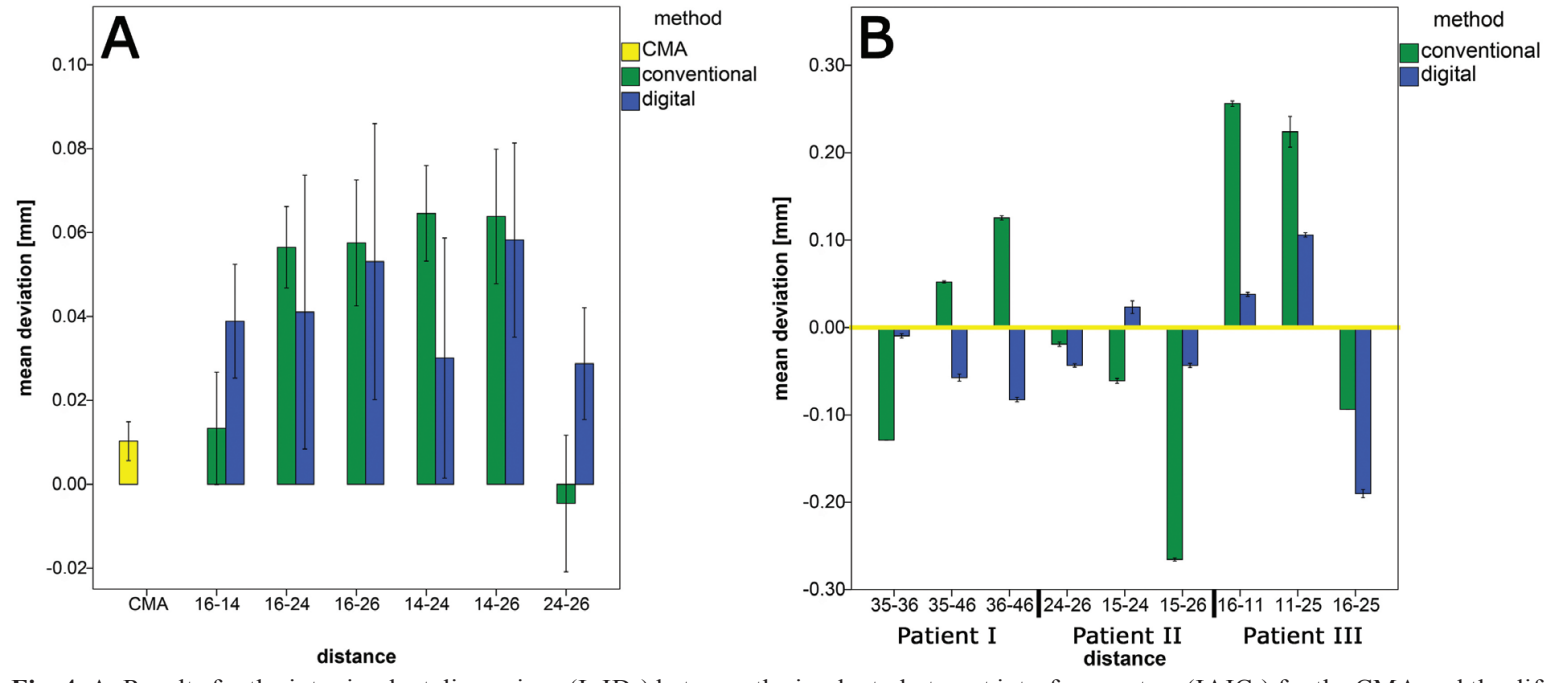
A: Results for the inter implant dimensions (InIDs) between the implant-abutment interface centers (IAICs) for the CMA and the different impression methods (*in vitro* part). B: Results for the inter implant dimensions (InIDs) for the pilot cases *in vivo* (the yellow zero line represents the CMA).

For a better overview, the results of the inter implant dimensions (InIDs) between the IAICs for the conventional and digital impression methods in the *in-vitro* setup are depicted in Fig. 4A. With the CMA method a trueness of 10,3 µm and a precision of 18 µm could be achieved.

The p-values for the InIDs within the conventional and digital impressions are presented in  Table 1. There were partially significant differences between the InIDs of the CMA and the conventional and digital impressions.

**Table 1:**
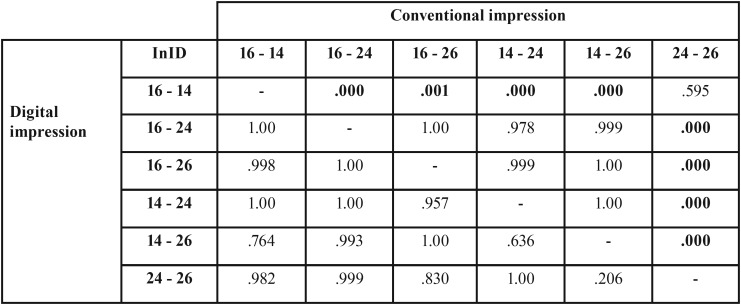
The *p*-values for the inter implant dimensions (InIDs) between the CMA and the conventional and the digital impressions (*p*-values <.05 are printed in bold type; implant positions FDI labeled).

The results for the three pilot cases in the *in-vivo* setup are presented in Fig. 4B. The hypothesis could be confirmed, the 3D-method reproduces the three-dimensional inter implant dimensions (InIDs) decisively better than the conventional or digital impression.

## Discussion

There are very few clinical studies available that address the accuracy of full arch implant impressions in patients (7,8), which reflects the high need for an appropriate method to assess the three-dimensional inter implant dimension directly in the patient’s mouth. Therefore, the purpose of this study was to develop a method to precisely assess the spatial orientation (inter implant dimension) of a minimum of three implants in the patient’s mouth for the *in-vivo* assessment of different impression methods (conventional and digital). The design of the IMM based on a clinical case was selected to represent a common clinical situation with and without angulated implants.

The novel nature of the presented method is the direct measurement of the implant position (model analogue) with the help of a custom made measuring aid with the CMM (Fig. 1F). Only a very small amount of impression material with a high final hardness was necessary to fix the CMA to the implant impression posts; therefore, other possible inaccuracies resulting from unavoidable dimensional changes of the impression material can be minimized (9,10). However, the use of a rigid resin material for fixation could be more accurate, but divergent axes of the impression posts make this impossible. To further reduce measurement and calculation errors, the dimensions of the laboratory analogues used in the measurement process were precisely measured with industrial computed tomography prior to the study (11).

This method, in combination with the high measuring precision of the CMM, allowed us to access the spatial orientation of implants with a trueness of 99.97 %. Although the mean absolute error was 10 µm, the method can be considered as an appropriate tool for the assessment of implant position in patients in a clinical setting.

The trueness obtained with the method described is much higher than the trueness achievable with cone beam computed tomography (140 µm) as described by (12). However, the latter technique allows for an assessment of the spatial orientation of the implants compared to the other oral components, such as teeth, which is not possible with the CMA method and is a clear shortcoming. On the other hand, the high X-ray exposure is regarded as a hindrance in clinical studies. Regarding optical scanning procedures using high precision industry scanners, instead of intraoral scanners, that deliver accuracies up to 7 µm, these systems are not suiTable for intraoral application.

A conventional impression or intraoral scan of the implants with the help of scanbodies and a common intraoral scanner is also decisively less precise. This observation is clearly reflected in our data, which are in good accordance with several other studies (13-15,5,16). As the scanning path used with IOS is known to influence the accuracy, we utilized the scanning path recommended by the manufacturer (17). The increasing inaccuracies of the teeth 24 and 26 could be explained due to the longer scanning path. Similar results were also reported by (18) and (19). These results were confirmed in the *in-vivo* part of the present study. In accordance to the *in-vitro* part, a clear increase in deviations can be seen with increasing recording length and scanning path. For the patient 1, the highest deviation could be observed between the implant in regio 36 and the implant in regio 46, this was likewise the longest scanning distance. Patient 3 also showed the greatest inaccuracies between the longest distance (16-25). In contrast to the in-vitro part, however, the digital impressions achieved partially results more precise.

For conventional impressions, a common impression material and method were used (20), this procedure corresponds to the usual clinical procedure in patients. This approach is especially common for the impression of angulated implants, which use the open tray technique for conventional impressions and could achieve the highest accuracy (21). Within conventional impressions, a mean overall value of 42 µm could be achieved. These results are in the range of the studies described in the literature (16). The more accurate results in the literature could be explained by a different reference system, which is similar to (6). In the *in-vitro* part of the present study, all the measurements were compared to the same coordinate system. Other studies could achieve more inaccurate results compared to the present study. This observation could be explained by the digitization of plaster casts and the resulting inaccuracies (22-24) or by the *in-vivo* conditions (8). Furthermore, the differences that occurred may be based on the difficultly of the comparison due to the many influencing factors within implant impressions (25).

Most of the available investigations used a best-fit algorithm to describe the deviations (21,19). Only a few authors described point-to-point measurements in a coordinate system, which are genuinely more precise (26). In another study on natural teeth (27), the measuring error was reduced to less than 15 µm for the longest transversal distance in the lower arch. In the present study, we were able to reduce the error for the longest distance to less than 13 µm. The highest standard deviation was observed between the implant in regio 16 and the 15° angulated implant in regio 24 implant (24 µm). This observation could be explained by the compression of the impression material that was used for fixation of the impression posts in the CMA during its removal from the implants. These results are in accordance with (28,29) and (30), where impressions of parallel implants also resulted in the highest accuracy.

Overall, the data presented here could show, that the developed method using a CMA is capable to assess the true three dimensional inter implant dimensions (spatial orientation) with a decisively higher trueness and precision than possible with a conventional or digital implant impression. However, the need for a custom made measuring aid for every single case is clear limitation to the method. The partially heterogeneous data show that a follow-up clinical study with a higher number of probands is necessary.
